# Multi-instance learning based lung nodule system for assessment of CT quality after small-field-of-view reconstruction

**DOI:** 10.1038/s41598-024-53797-4

**Published:** 2024-02-07

**Authors:** Yanqing Ma, Hanbo Cao, Jie Li, Mu Lin, Xiangyang Gong, Yi Lin

**Affiliations:** 1 Department of Radiology, Zhejiang Provincial People’s Hospital (Affiliated People’s Hospital, Hangzhou Medical College), Hangzhou, 310014 Zhejiang China; 2 Infervision Technology Co. Ltd., Beijing, 100010 China

**Keywords:** Computed tomography, Small-field-of-view, Multi-instance learning, Contrast-to-noise ratio, Cancer, Medical research

## Abstract

Small-field-of-view reconstruction CT images (sFOV-CT) increase the pixel density across airway structures and reduce partial volume effects. Multi-instance learning (MIL) is proposed as a weakly supervised machine learning method, which can automatically assess the image quality. The aim of this study was to evaluate the disparities between conventional CT (c-CT) and sFOV-CT images using a lung nodule system based on MIL and assessments from radiologists. 112 patients who underwent chest CT were retrospectively enrolled in this study between July 2021 to March 2022. After undergoing c-CT examinations, sFOV-CT images with small-field-of-view were reconstructed. Two radiologists analyzed all c-CT and sFOV-CT images, including features such as location, nodule type, size, CT values, and shape signs. Then, an MIL-based lung nodule system objectively analyzed the c-CT (c-MIL) and sFOV-CT (sFOV-MIL) to explore their differences. The signal-to-noise ratio of lungs (SNR-lung) and contrast-to-noise ratio of nodules (CNR-nodule) were calculated to evaluate the quality of CT images from another perspective. The subjective evaluation by radiologists showed that feature of minimal CT value (*p* = 0.019) had statistical significance between c-CT and sFOV-CT. However, most features (all with *p* < 0.05), except for nodule type, location, volume, mean CT value, and vacuole sign (*p* = 0.056–1.000), had statistical differences between c-MIL and sFOV-MIL by MIL system. The SNR-lung between c-CT and sFOV-CT had no statistical significance, while the CNR-nodule showed statistical difference (*p* = 0.007), and the CNR of sFOV-CT was higher than that of c-CT. In detecting the difference between c-CT and sFOV-CT, features extracted by the MIL system had more statistical differences than those evaluated by radiologists. The image quality of those two CT images was different, and the CNR-nodule of sFOV-CT was higher than that of c-CT.

## Introduction

Lung carcinoma is one of the most frequently diagnosed carcinomas and remains the leading cause of cancer-related mortality^1^, accounting for 18% of total cancer deaths worldwide in 2020^2^. Conventional computed tomography (c-CT) is a screening method that can reduce mortality rates compared to chest X-rays^3^, and is routinely used to evaluate lung nodules. In addition to detecting lung nodules, CT can accurately diagnose these nodules, facilitating the selection of better therapies^4^. Therefore, exploring CT reconstruction techniques is crucial to improve the display of fine structures of lung tissues and surrounding tissues, including the tumoral margin, pulmonary vessels, peripheral bronchus, and adjacent pleura, without increasing the radiation exposure^5^. It has been demonstrated that different CT reconstruction settings affect the diagnostic performance of lung nodules in a commercially available computer-aided diagnosis system^6^.

The approach of using a multi-instance learning (MIL) system to analyze lung nodules is an active field that helps doctors detect leaks and serves as a reference^7^. To maintain diagnostic performance and minimize excessive radiation, small-field-of-view reconstruction CT images (sFOV-CT) increase pixel density across airway structures and reduce partial volume effects^8^. Moreover, sFOV in CT are proved to be associated with higher spatial resolution and clearer images after subjective evaluation by radiologists. It has been demonstrated that reducing the reconstructed field of view (FOV) has a beneficial impact on increasing diagnostic image quality in CTA run-off examinations, regardless of vessel size^9^. Furthermore, a smaller and adjusted FOV or an increased matrix was well established to improve the spatial resolution in other part of body in CT^8^. The specific sFOV reconstruction can be easily achieved in most clinical CT scanners and does not require any additional hard- or software. The sFOV reconstruction approach also has been widely studied in the diffusion-weighted sequence in prostate cancer^10^, rectal carcinoma^11^, and other areas. Signal-to-noise ratio (SNR) and contrast-to-noise ratio (CNR) are frequently employed metrics for quantifying the grayscale variation between two different tissues. SNR was defined as the intensity ratio of image to background noise, and CNR was the signal difference between two tissues. To best of our knowledge, no prospective clinical study has investigated the influence of sFOV-CT in evaluating lung nodules compared to c-CT using a MIL-based lung nodule system and assessed their SNR and CNR. Therefore, the purpose of this study was to subjectively by radiologists and objectively by a MIL system to evaluate the difference between c-CT and sFOV-CT, and assessed the image quality by SNR and CNR.

## Materials and methods

### Study population

**Figure 1:**
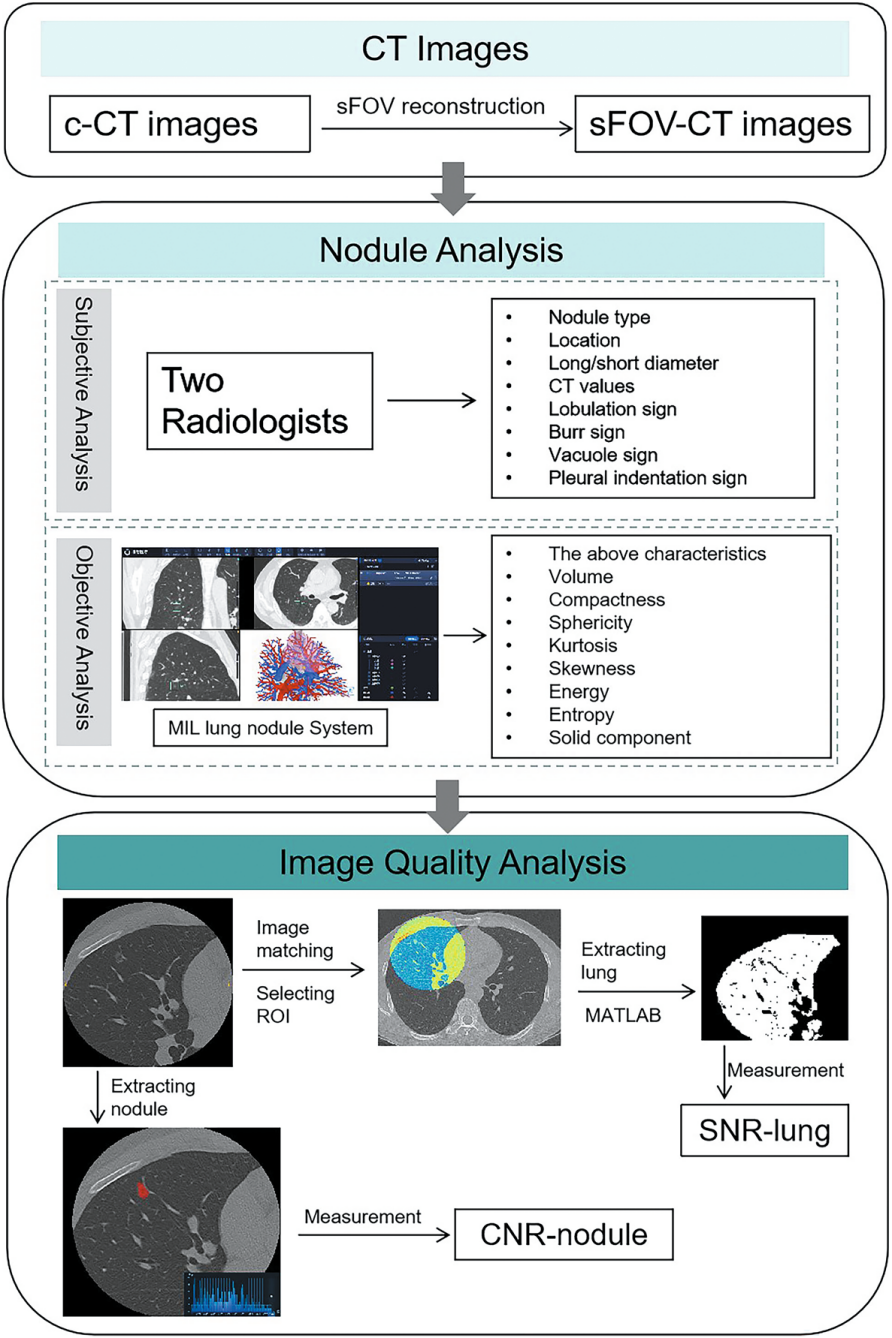
The flowchart of this study in comparison of the c-CT and sFOV-CT to analyze pulmonary nodules.

This retrospective study was approved by the institutional ethical committee of our hospital and informed consents were waived (No. 2022QT108). A total number of 303 patients were collected in this study between July 2021 to March 2022, and the flowchart of study is shown in Fig. 1. The inclusion criteria were as follows: (1) patients who had an isolated lung nodule with a CT-displayed long diameter between 5 to 30 mm; (2) patients who had no history of other malignancies; (3) patients who underwent CT examinations using the same CT scanner and protocol; and (4) both subjective analysis by radiologist and objective analysis by a MIL system were performed for all patients. 303 patients were screened using the following exclusion criteria, such as: (1) patients who had other lesions surrounding the target lung nodule, including exudation (n = 37), strips (n = 56), pulmonary bullae (n = 14), emphysema (n = 21), and so on (n = 17); and (2) patients with motion (n = 19) or respiratory artifacts (27) in CT images. Finally, the image characteristics and image quality of c-CT and sFOV-CT in 112 patients was subjectively by radiologist and objectively by a MIL system compared.

### Conventional CT examination and analysis

In this retrospective study, all 112 patients underwent CT examinations using a 320-slice scanner (Aquilion ONE 320, TOSHIBA, Japan). Prior to scanning, patients were trained to control their breathing depth and frequency and were positioned in a supine position. The scanning range extended from the tip to the base of the lung. For c-CT, the following parameters were used: tube voltage of 120 kV, tube current of 250 mA, collimation of 0.5 × 64, matrix of 512 × 512, pitch of 1.484, FOV of 400 mm, slice thickness and interval of 1 mm, and window center and width of − 550 Hu and 1600 Hu, respectively. For sFOV-CT, the reconstruction parameters were a slice thickness and interval of 1 mm, an FOV of 150–160 mm, and a standard reconstruction algorithm.

Two radiologists, with 7 and 10 years of experience, evaluated the c-CT and sFOV-CT images together without knowledge of the pathological results. Any differences in their evaluation of the CT characteristics were resolved through discussion and consensus. The characteristics of c-CT selected for statistical analysis included the location (upper/middle/lower lobe of right lung, upper/middle lobe of left lung), type of lung nodule (pure ground glass nodule, pGGO; mixed ground glass nodule, mGGO; solid pulmonary nodule, SPN), shape sign (burr, lobulation, vacuole, and pleural indentation sign), size (CT-displayed long/short diameter), maximum CT value (CT-maximum), minimum CT value (CT-minimum), mean CT value (CT-mean), and the standard deviation of CT value (CT-SD).

### The analysis of MIL system

The MIL system (InferRead CT target Reconstruction, Version 0.0.1) was used to analyze c-CT (c-MIL) and sFOV-CT (sFOV-MIL) images. The MIL system not only analyzed the features subjectively evaluated by radiologists but also calculated additional indicators, including the volume of the lung nodule, proportion of solid component, compactness, sphericity, kurtosis, skewness, energy, and entropy. MIL is a machine learning method where labels are assigned to bags rather than individual instances, which is different from conventional machine learning. It can benefit from a clinical practical standpoint, as it would theoretically enable radiologists to benefit from a large amount of unlabeled or irrelevantly labels.

The volume of the lung node was automatically measured by the MIL system, while the proportion of solid component was quantified according to the proportion of solid component in lung nodule based on a threshold of − 145 Hu. Compactness and sphericity were used to measure how closely the tumor shape resembled a sphere and the roundness of the tumor region relative to a sphere, respectively. Kurtosis measured the “peakness” of the distribution of values in the image, and skewness measured the asymmetry of the distribution of values about the mean value. Energy measured the magnitude of voxel values in an image, while entropy measured the average amount of information required to encode the image values, indicating the randomness in the image values. Further details of the parameters used in the MIL system can be found in the Supplementary Material.

### Image quality assessment

To evaluate image quality, two metrics of signal to noise ratio in the lung (SNR-lung) and contrast to noise ratio in the nodule (CNR-nodule) were calculated using MATLAB software (MathWorks Inc, Natick, Massachusetts, USA). SNR-lung was calculated as the ratio of mean value to standard deviation (SD) in the same range of region of interest (ROI). The equation used for calculating CNR-nodule was: (mean_nodule_ − mean_lung_)/SD_lung_. The specific procedure is described in detail in the Supplementary Material.

### Statistical analysis

The Shapiro–Wilk normality test was used to assess the normal distribution of all data. The normally distributed continuous variables were analyzed using Student’s *t*-test and presented as means ± SD. Non-normally distributed continuous variables were analyzed using the Mann–Whitney *U* test and presented as median (inter-quartile range). Categorical variables were evaluated using Pearson Chi-square test or Fisher’s exact test. Statistical analyses were conducted using software of SPSS (IBM SPSS Statistics, Version 26) and MedCalc (MedCalc Software Ltd, Version 20.100). A two-tailed *p*-value < 0.05 was considered statistically significant.

### Institutional review board statement

This retrospective study was approved by the Medical Ethics Committee of Zhejiang Provincial People’s Hospital (No.2022QT108). All procedures were performed in accordance with the 1975 Declaration of Helsinki and its later amendments.

### Informed consent statement

The informed consent was waived for this retrospective study by the Medical Ethics Committee of Zhejiang Provincial People’s Hospital (No. 2022QT108).

## Results

### The subjective comparison of c-CT and sFOV-CT performance

**Figure 2:**
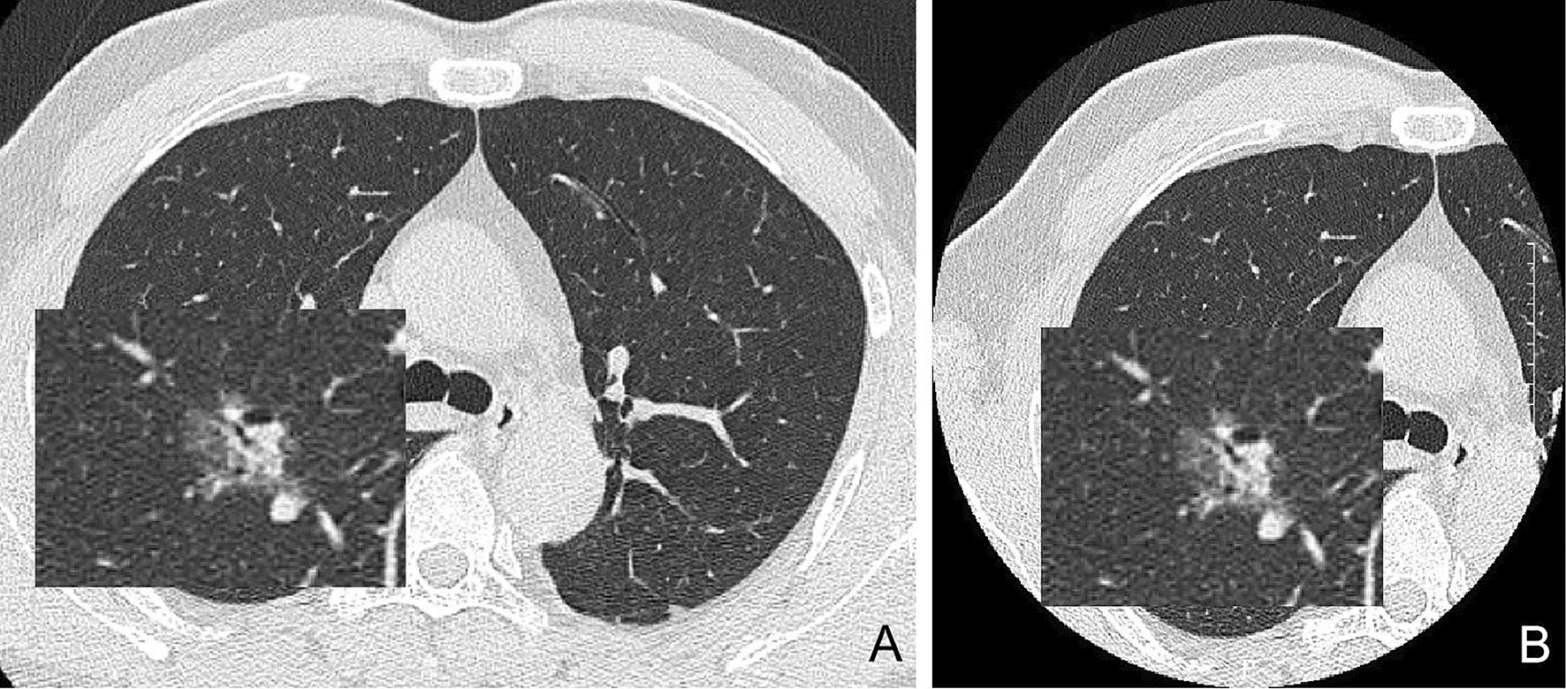
There was little difference between the c-CT and sFOV-CT images after subjective evaluation by radiologists.

Two radiologists manually detected the characteristics of lung nodules in c-CT and sFOV-CT images. The Mann–Whitney *U* test revealed a statistical difference in the characteristics of CT-minimum (*p* = 0.019), while no statistically significant difference was found in the characteristics of CT-displayed short/long diameter and CT-maximum/mean/SD (*p* = 0.059–0.793). Furthermore, after the Pearson Chi-square test, the characteristics of location, type, lobulation sign, burr sign, vacuole sign, and pleural sign also had no statistical significance (*p* = 0.639–1.000). As shown in Fig. 2, the nodules in c-CT and sFOV-CT images differed little by subjective evaluation. The specific data of CT characteristics from two radiologists was listed in the Supplementary Material.

### The comparison of c-MIL and sFOV-MIL performance

The characteristics of nodule type, location, volume, CT-mean, and vacuole sign showed no statistical significance, while significant differences were observed in the variables of long diameter (*p* = 0.001), short diameter (*p* = 0.001), CT-maximum (*p* < 0.001), CT-minimum (*p* < 0.001), CT-SD (*p* < 0.001), compactness (*p* < 0.001), sphericity (*p* < 0.001), kurtosis (*p* < 0.001), skewness (*p* < 0.001), energy (*p* < 0.001), entropy (*p* < 0.001), proportion of solid component (*p* < 0.001), lobulation (*p* = 0.008), burr sign (*p* = 0.012), and pleural (*p* = 0.031) sign (Table 1).

**Table 1:** The comparison of c-MIL and sFOV-MIL.

	c-MIL	sFOV-MIL	*p*
Type (N)			0.476
pGGO	45 (40.2%)	37 (33.0%)	
mGGO	59 (52.7%)	68 (60.7%)	
SPN	8 (7.1%)	7 (6.3%)	
Location (N)			1.000
Upper light lung	40 (35.7%)	39 (34.8%)	
Middle right lung	8 (7.1%)	8 (7.1%)	
Lower right lung	18 (16.1%)	18 (16.1%)	
Upper left lung	26 (23.2%)	27 (24.1%)	
Lower left lung	20 (17.9%)	20 (17.9%)	
Size			
Long diameter (mm)	11.100 (8.925 to 16.725)	8.700 (6.900 to 15.575)	0.001
Short diameter (mm)	9.000 (7.000 to 12.200)	7.150 (5.750 to 11.475)	0.001
Volume (mm^3^)	381.560 (193.463 to 1089.553)	300.315 (150.113 to 1223.965)	0.266
CT value			
CT-maximum (Hu)	− 47.000 (− 173.500 to 80.750)	276.500 (76.000 to 395.750)	< 0.001
CT-minimum (Hu)	− 750.500 (− 795.750 to 674.750)	− 796.500 (− 843.750 to 726.500)	< 0.001
CT-mean (Hu)	− 565.500 (− 636.250 to 435.750)	− 532.500 (− 614.500 to 369.250)	0.056
CT-SD	133.370 (103.030 to 165.730)	181.749 (158.432 to 223.166)	< 0.001
Texture			
Compactness	0.033 (0.026 to 0.039)	0.040 (0.037 to 0.042)	< 0.001
Sphericity	0.733 (0.628 to 0.807)	0.830 (0.780 to 0.860)	< 0.001
Kurtosis	− 0.840 (− 1.100 to 0.280)	− 0.025 (− 0.615 to 0.720)	< 0.001
Skewness	0.300 (0.110 to 0.630)	0.670 (0.400 to 0.988)	< 0.001
Energy	696,631,322.500 (308,851,215.000 to 1,695,575,343.000)	211,807,408.500 (114,155,253.300 to 697,188,246.800)	< 0.001
Entropy	4.518 (4.181 to 4.850)	5.081 (4.808 to 5.349)	< 0.001
Solid component %	0.640 (0.000 to 6.600)	5.795 (1.403 to 16.075)	< 0.001
Shape sign (N)			
Lobulation	42 (37.5%)	24 (21.4%)	0.008
Burr	35 (31.3%)	19 (17.0%)	0.012
Vacuole	6 (5.4%)	7 (6.3%)	0.775
Pleural indentation	14 (12.5%)	5 (4.5%)	0.031

**Figure 3:**
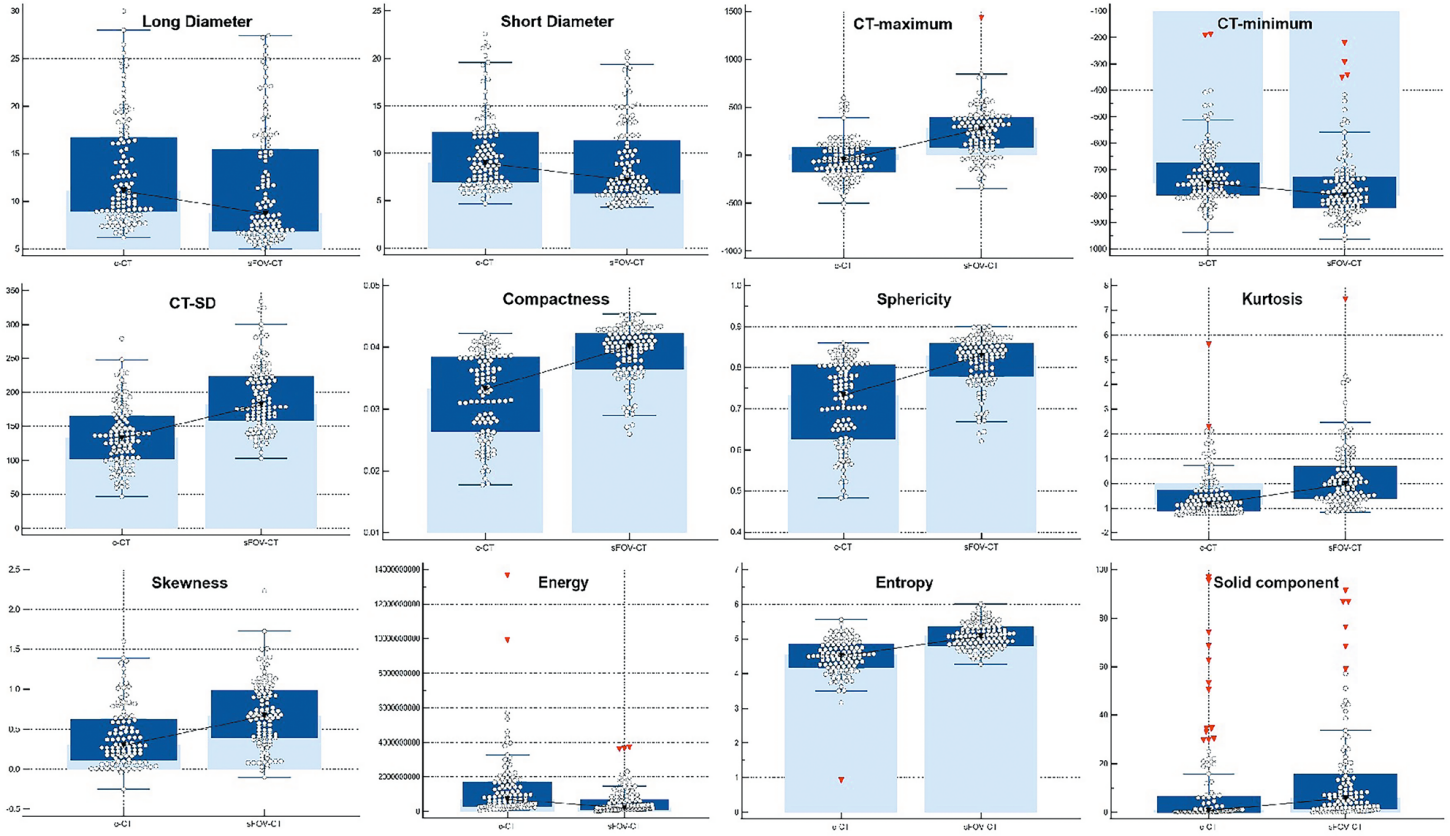
The Box-and-Whisker plots showed the significant difference of nodule characteristics of long/short diameter, CT-maximum/minimum/SD, compactness, sphericity, kurtosis, skewness, energy, entropy, and solid component.

**Figure 4:**
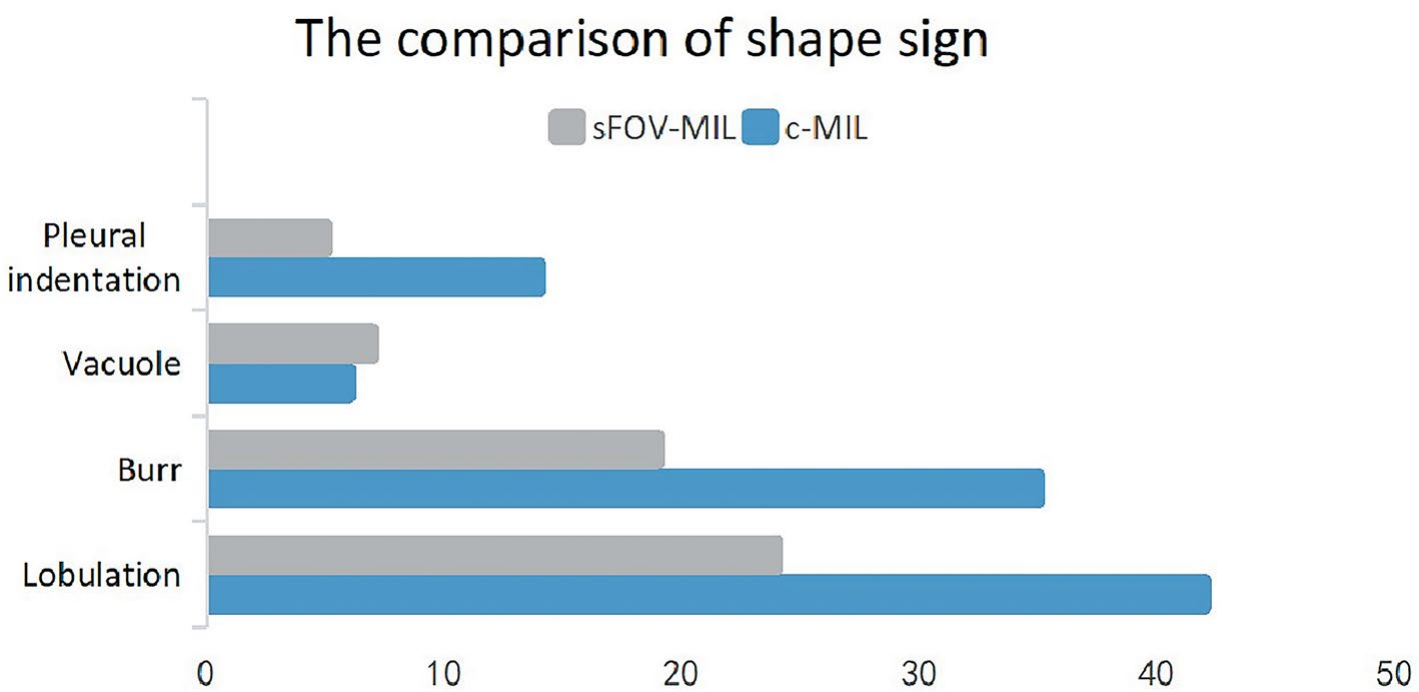
The bar graph showed the shape sign difference of pulmonary nodule by MIL system.

The median values of long and short diameter of nodules from sFOV-MIL were 8.700 (6.900–15.575) and 7.150 (5.750–11.475), respectively, which were significantly smaller than those from c-MIL, with the median values of 11.100 (8.925–16.725) and 9.000 (7.000–12.200), respectively. The CT-maximum from sFOV-MIL was larger than that from c-MIL (276.500, 76.000–395.750 vs. − 47.000, − 173.500 to 80.750), while the CT-minimum from sFOV-MIL was smaller than that from c-MIL (− 796.500, − 843.750 to 726.500 vs. − 750.500, − 795.750 to 674.750). The median of CT-SD from sFOV-MIL was 181.749 (158.432–223.166), larger than that from c-MIL. The median value of compactness, sphericity, kurtosis, skewness, and entropy from sFOV-MIL were larger than those from c-MIL, while the energy was relative smaller than those from c-MIL. The proportion of solid component from sFOV-MIL was larger than that from c-MIL (5.795, 1.403–16.075 vs. 0.640, 0.000–6.600). Figure 3 illustrates the deviation between c-MIL and sFOV-MIL in evaluating nodules using Box-and-Whisker plots. More cases analyzed by c-MIL detected the characteristics of lobulation sign, burr sign, and pleural indentation sign (Fig. 4).

### The comparison of image quality

**Figure 5:**
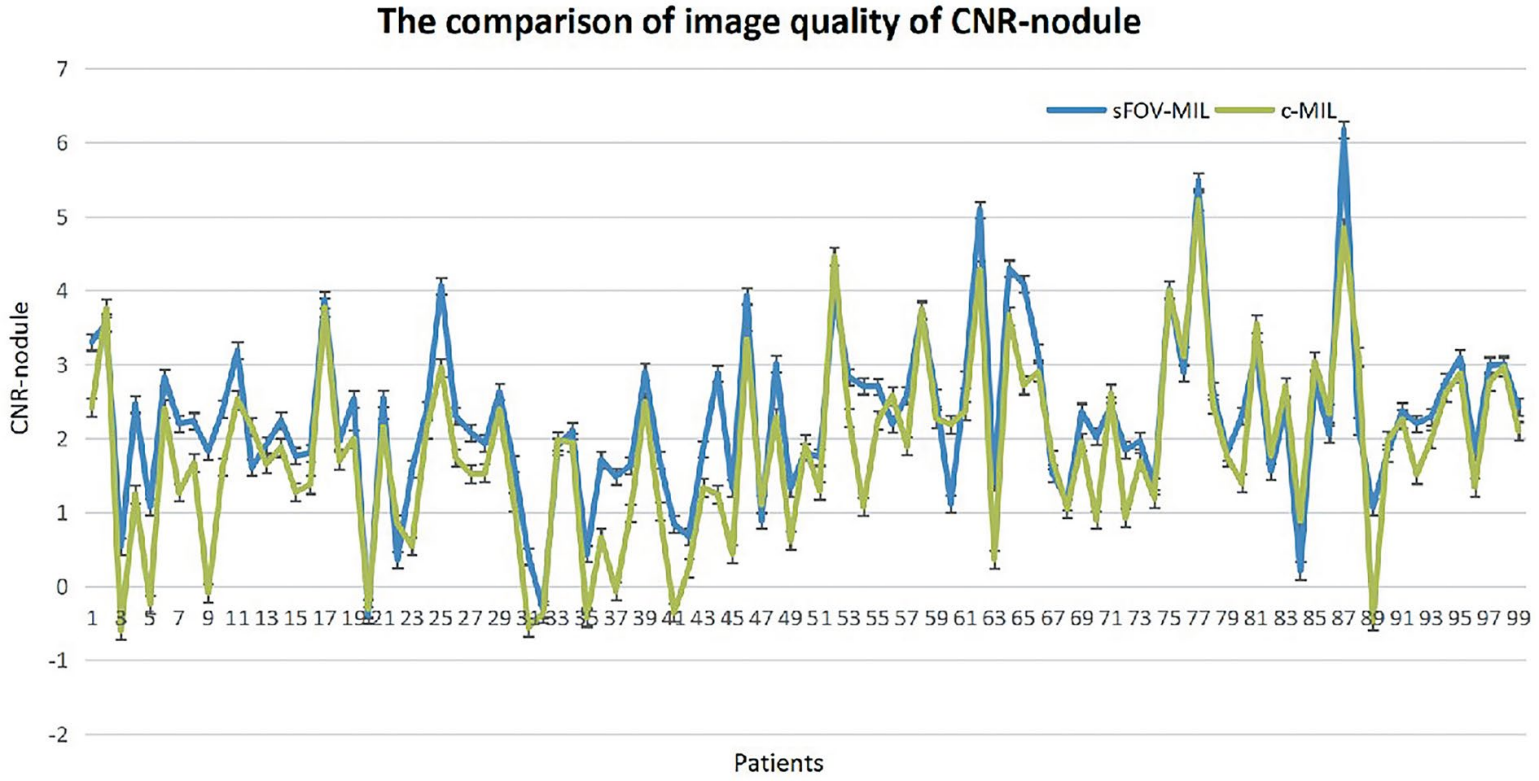
The CNR-nodule from c-MIL and sFOV-MIL was statistically different, and the CNR-nodule of sFOV-MIL was higher than that of c-MIL.

The indicators of SNR-lung and CNR-nodule were calculated to evaluate the quality of c-CT and sFOV-CT images, and their non-normal distribution was confirmed by the Shapiro–Wilk test (*p* < 0.05). The method of Mann–Whitney *U* test was used to analyzed the data, which revealed no statistical significance for SNR-lung (*p* = 0.913). However, CNR-nodule statistically differed (*p* = 0.007), and the median CNR-nodule in sFOV-CT was 2.203 (1.620–2.793), which was larger than that of c-CT with median of 1.850 (1.027–2.558), as shown in Fig. 5.

## Discussion

Smaller FOVs in CT images are often associated with clearer and higher resolution images^12^. Studies have reported that a small FOV reconstruction in CT angiography of the lower extremities significantly improved image quality compared to a large FOV reconstruction covering two legs^9^. In order to explore a better approach to presenting pulmonary nodules, we applied an sFOV reconstruction algorithm to c-CT images and compared their difference statistically. As far as we know, there are currently no available articles that have subjectively been reviewed by radiologists and objectively evaluated by software to assess the quality of conventional and sFOV reconstruction CT images. According to the results from previous research, different reconstruction parameter settings affected the performance of a commercially available deep learning based lung nodule CAD system, objectively^6^. Other studies have shown that the reduction of FOV increased the accuracy of airway wall thickness quantification by radiologists, subjectively^8^. So, there may be some fascinating findings to evaluate the performance of lung nodules in both conventional and sFOV reconstruction CT images. In terms of subjective prediction, we observed that the most CT characteristics, except for CT-minimum, had no statistical significance between c-CT and sFOV-CT images. Therefore, we concluded that the sFOV-CT images would not affect the subjective evaluation of CT characteristics of lung nodules, including CT values and shape features, by radiologists’ visual assessment. Previous studies subjectively evaluated and scored the images by radiologists found that image quality did not improve in reduced FOV of 160 mm in c-CT^12^, which is consistent with our findings.

The use of smaller or adjusted FOV in CT imaging has been shown to improve spatial resolution in cadaveric lung studies^13^. Shenshadri et al. found that CT examinations on lung phantom and subjects with reconstruction at 10–30 cm FOV using a medium-smooth kernel and reduced reconstruction FOV would minimally increase the sensitivity to detect the differences of airway dimensions in asthma^8^. Furthermore, reducing the size of FOV to 160 mm in high-resolution CT and 80 mm in ultra-high-resolution CT can improve diagnostic imaging quality^12^. In our study, we objectively compared the manifestation of c-CT and sFOV-CT using a MIL system. Contrary to the subjective assessments by radiologists, most features showed statistical differences except for type, location, volume, CT-mean, and lobulation sign. This may be because MIL system recognizes characteristics of lung nodules often overlooked by visual inspection alone. Previous studies have reported that computer-aid diagnosis system improve radiologists’ ability to detect pulmonary nodules smaller than 5 mm, which are often missed by visual inspection alone^14^, and have the potential to improve accuracy in diagnosing pulmonary nodules^15^. Therefore, our results suggest that the sFOV-CT is objectively different from the c-CT, despite being easily overlooked in visual assessment.

To detect the different quality of two types of CT images. We quantified the image quality using SNR-lung and CNR-nodule. CNR is an important tool for evaluating image quality, which was established on a contrast^16^. We found that the CNR-nodule of c-CT and sFOV-CT were statistically different, with sFOV-CT having a higher CNR-nodule than of c-CT (median, inter-quartile: 2.203, 1.620–2.793 vs. 1.850, 1.027–2.558). This is consistent with previous finding by Harder FN, who showed that reduced FOV acquisition in diffusion-weighted MRI can provide higher image quality and apparent CNR values^17^. A previous study on rectal carcinoma also found that reduced FOV diffusion-weighted imaging sequences provided higher CNR and better lesion conspicuity than full FOV ones^18^. Therefore, the sFOV reconstruction of pulmonary nodules based on conventional CT images can be beneficial, particularly if additional radiation dose is to be avoided.

There are several limitations to this study that need to be addressed. First of all, the FOV range of the sFOV reconstruction in our study was between the range from 150 to 160 mm due to the difference in body shape. Secondly, our study was focused on evaluating image quality and CT characteristics rather than evaluating the impact on clinical decision-making. Further studies are necessary to confirm and evaluate the diagnostic difference between c-CT and sFOV-CT images. Thirdly, we used a standard algorithm technique to reconstruct sFOV-CT images. Other reconstruction techniques, such as sharp, smooth, and volume algorithms, and different scanning machines may produce disparity, which requires further exploration.

## Conclusions

In conclusion, we have demonstrated that MIL system is better equipped to detect the difference between c-CT and sFOV-CT than subjective evaluation by radiologists. Our findings show that their image quality is statistically different, and the CNR-nodule of sFOV-CT is higher than that of c-CT. Therefore, sFOV reconstruction based on c-CT images is conducive to improve the conspicuity of pulmonary nodules.

### Supplementary Information

Supplementary Information.

## Data Availability

The datasets generated during and/or analyzed during the current study are available from the corresponding author on reasonable request.
