# Supplementary material for: Multi-instance learning based lung nodule system for assessment of CT quality after small-field-of-view reconstruction
Source: Sci Rep. 2024 Feb 7;14:3109. doi: 10.1038/s41598-024-53797-4 (PMC10850475; doi:10.1038/s41598-024-53797-4)
Supplement: Supplementary file 1 — Supplementary Information. [file 41598_2024_53797_MOESM1_ESM.doc]

**Supplementary Material**

1. **The characteristics from MIL system: including type of nodule, location, short/long diameter, volume of lung nodule, maximum/minimal/mean/standard deviation value of CT, proportion of solid component, compactness, sphericity, kurtosis, skewness, energy, and entropy.**

**Type of nodule: The lung nodules were divided into three types, including pure ground glass opacity (pGGO), mixed ground glass opacity (mGGO), solid pulmonary nodule (SPN), according to the percentage of solid component in the nodule.**

**Location: The location of nodule was divided into the upper right lobe, middle right lobe, lower right lobe, upper left lobe, and lower left lobe.**

**Volume of lung nodule: The lung nodule was automatically segmented, then the volume of lung nodule was automatically measured by the software of InferRead CT target Reconstruction (Version 0.0.1).**

**Proportion of solid component: The proportion of solid component was quantified according to the proportion of solid component in lung nodule based on a threshold of -145 Hu.**

**Compactness: Similar to Sphericity, Compactness is a measure of how compact the shape of the tumor is relative to a sphere (most compact). It is therefore correlated to Sphericity and redundant. It is provided here for completeness. The value range is 0<compactness≤1/6π, where a value of 1/6π indicates a perfect sphere. By definition, compactness = 1/6π √sphericity3.**


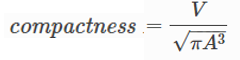


**Sphericity:** Sphericity is a measure of the roundness of the shape of the tumor region relative to a sphere. It is a dimensionless measure, independent of scale and orientation. The value range is 0<sphericity≤1, where a value of 1 indicates a perfect sphere (a sphere has the smallest possible surface area for a given volume, compared to other solids).


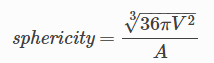


**Kurtosis:** Where μ4 is the 4th central moment. Kurtosis is a measure of the ‘peakedness’ of the distribution of values in the image ROI. A higher kurtosis implies that the mass of the distribution is concentrated towards the tail(s) rather than towards the mean. A lower kurtosis implies the reverse: that the mass of the distribution is concentrated towards a spike near the Mean value.


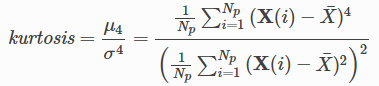


**Skewness:** Where μ3 is the 3rd central moment. Skewness measures the asymmetry of the distribution of values about the Mean value. Depending on where the tail is elongated and the mass of the distribution is concentrated, this value can be positive or negative.


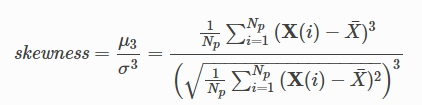


**Energy:** Here, **c** is optional value, defined by voxel Array Shift, which shifts the intensities to prevent negative values in **X**. This ensures that voxels with the lowest gray values contribute the least to Energy, instead of voxels with gray level intensity closest to 0. Energy is a measure of the magnitude of voxel values in an image. A larger values implies a greater sum of the squares of these values.


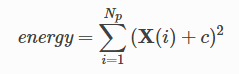


**Entropy:** Here,**ϵ** is an arbitrarily small positive number (≈2.2×10^−16^). Entropy specifies the uncertainty/randomness in the image values. It measures the average amount of information required to encode the image values.


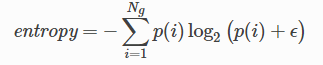


1. **The MIL system**

The lung nodule diagnosis system (InferRead CT Target Reconstruction, Version 0.0.1) can automatically detected the nodules and measure their parameters in this study. The algorithm of this system based on multi-instance learning (MIL) to establish a training set with classification labels by taking multi-example packages as training units, to establish a training set with classification labels. Based on the training unit obtained by MIL, the backbone model adopts residual convolutional neural network (ResNet18) to extract features from K examples. Compared with traditional convolutional neural network, ResNet has great advantages in solving the problem of gradient disappearance or gradient explosion degradation caused by the network layer increasing. At the same time, the structure of ResNet can accelerate the training of neural network very quickly, and the accuracy of the model also has a better performance. The following is the structure diagram of ResNet 34-layer model:


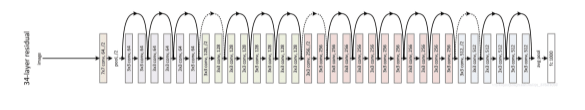


The images of each sample package were extracted by feature fusion of residual convolution neural network 2D ResNet18 and attention-based deep MIL (attention-based deep MIL). All features were attention-weighted, and then the variable-length sequence images were classified and analyzed. For patients with pulmonary nodules, we designed a deep learning network based on 3D ResNet to automatically segment pulmonary nodules based on the lung segmentation model obtained from the internal lung database training of Inference technology. Then, the CT value range related to the image signs of nodules was retained in the images of the lung region, which was extracted and filled into cuboids of specific size. Finally, combined with the original CT image of lung segmentation image and space cuboids within a certain HU range, the original CT image was input into the 3D ResNet network for feature extraction and analysis, and the model output four-dimensional vector results.

1. **The normality test and subjective measurement of CT characteristics**

Table E1. The normality test of CT characteristics

|  | **statistics** | **df** | ***p*** |
| --- | --- | --- | --- |
| **Long diameter** | **0.909** | **224** | **0.000** |
| **Short diameter** | **0.874** | **224** | **0.000** |
| CT-maximum | **0.986** | **224** | **0.030** |
| CT-minimum | **0.974** | **224** | **0.000** |
| CT-mean | **0.960** | **224** | **0.000** |
| CT-SD | **0.967** | **224** | **0.000** |

**The results of normality test of characteristics from conventional and small-field-of-view reconstruction CT images.**

Table E2. The subjective CT analysis of nodules

|  | c-CT | sFOV-CT |
| --- | --- | --- |
| Location (RU/RM/RL/LU/LL) | 40/8/18/27/19 | 40/8/19/26/19 |
| Type (pGGO/mGGO/SPN) | 30/71/11 | 30/71/11 |
| Size |  |  |
| Long diameter | 11.250(7.975-14.525) | 10.700(8.250-15.175) |
| Short diameter | 7.800(6.300-10.375) | 8.150(6.500-10.100) |
| CT values |  |  |
| CT-maximum | -10.500(-200.250-180.750) | 55.000(-164.500-243.750) |
| CT-minimum | -848.000(-962.250--694.750) | -891.000(-1133.000--748.250) |
| CT-mean | -470.955(-578.053--303.240) | -470.685(-589.583--331.615) |
| CT-SD | 173.130(135.028-215.675) | 192.245(145.258-236.745) |
| Shape sign |  |  |
| Lobulation | 33 | 34 |
| Burr | 12 | 13 |
| Vacuole | 9 | 11 |
| Pleural indentation | 14 | 15 |

The specific data of c-CT and sFOV-CT characteristics by two radiologists. As the characteristics were non-normal distributed, so they were displayed as median (inter-quartile range). RU: upper right lung, RM: middle right lung, RL: lower right lung, LU: upper left lung, LL: lower left lung; pGGO: pure ground glass opacity, mGGO: mixed ground glass opacity, SPN: solid pulmonary nodule; c-CT: conventional CT; sFOV-CT: small-field-of-view CT.

1. **The assessment of image quality**

For the evaluation of image quality, two metrics of SNR-lung and CNR-nodule were calculated. The SNR-lung was calculated as the ratio of mean value to standard deviation (SD) in the ROI:


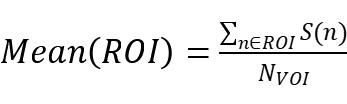
 (1)


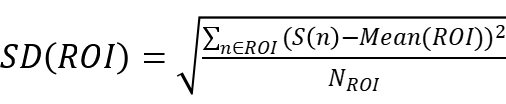
 (2)


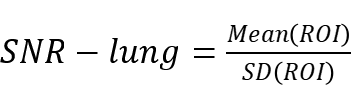
 (3)

where Mean(ROI) and SD(ROI) are the mean and standard deviation values of the region of interest, respectively. N_ROI_ is the number of voxels in the ROI. In this study ROI is the lung parenchyma as shown in sFigure 1.

The contrast to noise ratio (CNR) is a measure of the signal level in the presence of noise given by:


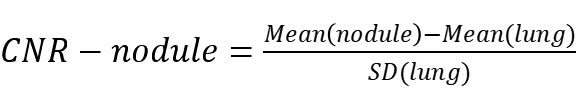
 (4)

where mean(lung) and SD(lung) were measured in lung parenchyma as in equation(1-3). And the mean(nodule) is the mean value of the nodule.


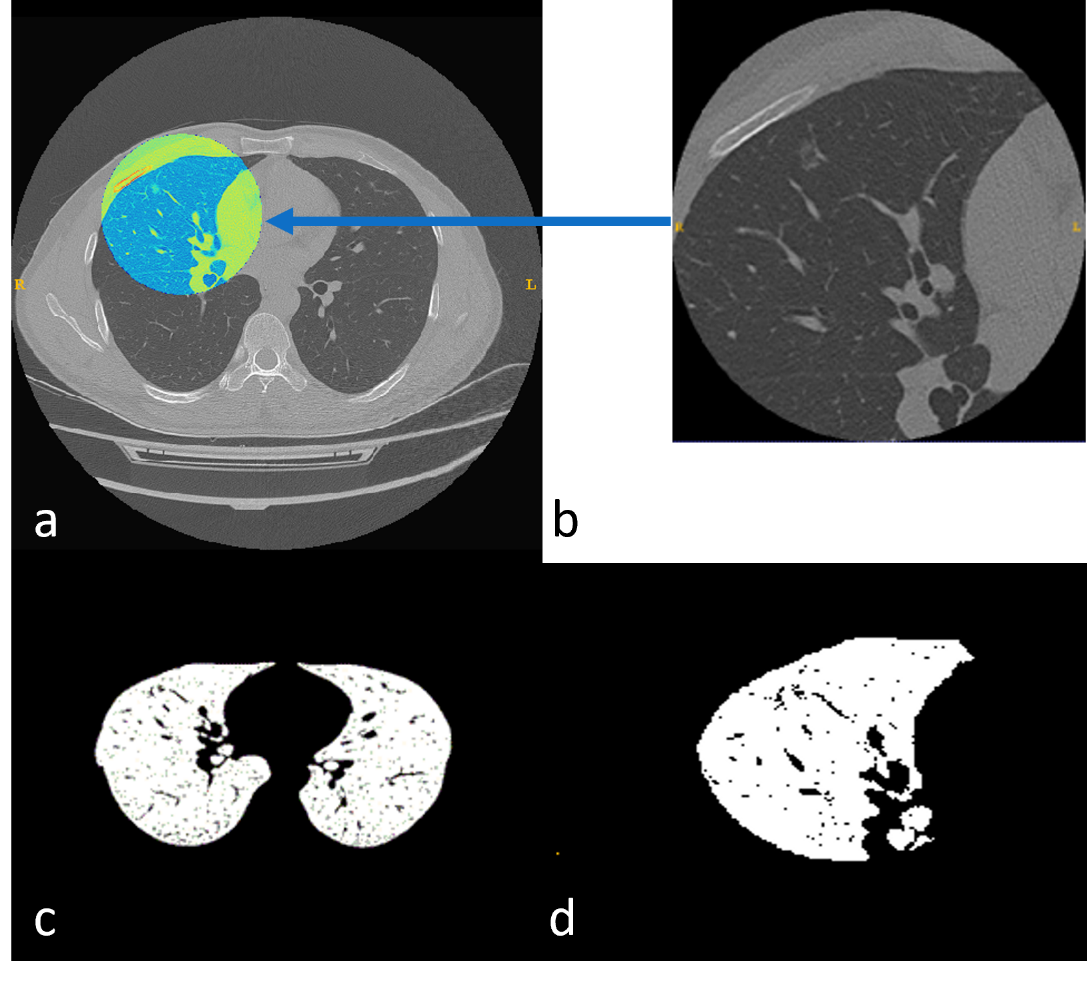


sFigure 1. Extraction of lung parenchyma after image matching in software of MATLAB. (a) The original CT image with target reconstruction area overlaid. (b) The s-FOV CT image. (c, d) The lung parenchyma was extracted with threshold.
